# Balance in single-limb stance after surgically treated ankle fractures: a 14-month follow-up

**DOI:** 10.1186/1471-2474-7-35

**Published:** 2006-04-05

**Authors:** Gertrud Nilsson, Eva Ageberg, Charlotte Ekdahl, Magnus Eneroth

**Affiliations:** 1 Primary Healthcare Research Department, Lund University Hospital, Lund, Sweden; 2 Department of Health Sciences, Division of Physiotherapy, Lund University, Lund, Sweden; 3 Department of Orthopaedics, Lund University Hospital, Lund, Sweden

## Abstract

**Background:**

The maintenance of postural control is fundamental for different types of physical activity. This can be measured by having subjects stand on one leg on a force plate. Many studies assessing standing balance have previously been carried out in patients with ankle ligament injuries but not in patients with ankle fractures. The aim of this study was to evaluate whether patients operated on because of an ankle fracture had impaired postural control compared to an uninjured age- and gender-matched control group.

**Methods:**

Fifty-four individuals (patients) operated on because of an ankle fracture were examined 14 months postoperatively. Muscle strength, ankle mobility, and single-limb stance on a force-platform were measured. Average speed of centre of pressure movements and number of movements exceeding 10 mm from the mean value of centre of pressure were registered in the frontal and sagittal planes on a force-platform. Fifty-four age- and gender-matched uninjured individuals (controls) were examined in the single-limb stance test only. The paired Student *t*-test was used for comparisons between patients' injured and uninjured legs and between side-matched legs within the controls. The independent Student *t*-test was used for comparisons between patients and controls. The Chi-square test, and when applicable, Fisher's exact test were used for comparisons between groups. Multiple logistic regression was performed to identify factors associated with belonging to the group unable to complete the single-limb stance test on the force-platform.

**Results:**

Fourteen of the 54 patients (26%) did not manage to complete the single-limb stance test on the force-platform, whereas all controls managed this (p < 0.001). Age over 45 years was the only factor significantly associated with not managing the test. When not adjusted for age, decreased strength in the ankle plantar flexors and dorsiflexors was significantly associated with not managing the test. In the 40 patients who managed to complete the single-limb stance test no differences were found between the results of patients' injured leg and the side-matched leg of the controls regarding average speed and the number of centre of pressure movements.

**Conclusion:**

One in four patients operated on because of an ankle fracture had impaired postural control compared to an age- and gender-matched control group. Age over 45 years and decreased strength in the ankle plantar flexors and dorsiflexors were found to be associated with decreased balance performance. Further, longitudinal studies are required to evaluate whether muscle and balance training in the rehabilitation phase may improve postural control.

## Background

Fractures involving the ankle are one of the most common injuries of the lower extremity with an incidence of 107 fractures per 10^5 ^person-years [1]. Fracture incidence in men is higher in younger ages (20–35 years) while the incidence in women increases after the age of fifty [2]. The treatment recommended for displaced ankle fractures is open reduction and internal fixation [3,4]. Adequate reduction with congruency of the joint has been reported as one of the most important predictors of a good end result [5-8]. Inadequate reduction may lead to osteoarthritis, which is also important for subjective outcome [5,7-9]. Physical outcome such as muscle strength [10], ankle range of motion [9,10], standing balance and the experience of perceived functional ankle instability has also been reported to influence subjective outcome [10].

The maintenance of postural control, defined as controlling the body's position in space for the dual purposes of stability and orientation, is fundamental for different types of physical activities. Increased sway area, measured by using a sway meter that measured displacement of the body at the level of the waist in 30-second periods, is reported as one of the predictors of sustaining a fracture in both men and women [11]. Balance training, defined as the ability o maintain the position of centre of body mass within the stability limits, has been shown to improve postural control [12-15] and to prevent recurrent injuries [16]. Postural control is often measured by having subjects stand on a computerized force-platform. Centre of pressure (CP) movements (amplitude and speed) are measured from the ground reaction forces. The single-limb stance test on a force-platform is a reliable method [17,18] and is commonly used for evaluation of functional instability and ankle ligament injuries [14,19-22]. Impaired postural control with increased amplitude and increased CP movements has been reported in patients with ligament injuries of the ankle [20,23]. To our knowledge no equivalent investigations have been carried out in patients with ankle fractures. In a previous study of the same patient group we found that decreased balance measured as standing on one leg on the floor for 60 seconds showed a strong relation to self-reported decreased function 14 months postoperatively [10]. The purpose of the present study was to evaluate whether patients operated on because of an ankle fracture had impaired postural control, measured by single-limb stance on a force-platform, compared to an uninjured age- and gender-matched control group.

## Methods

### Patients

Fifty-four patients, 31 men and 23 women, consecutively operated on were asked to participate and included in the study [10]. The median age at the time of injury was 36 years for men (Interquartile range, (IQR) 28) and 51 for women (IQR 15). Before surgery all fracture types were classified showing 37 supination-eversion (SE) injuries (5 SE II, 4 SE III, 28 SE IV), 2 supination-adduction (SA) injuries (2 SA II), 13 pronation-eversion (PE) injuries (7 PE III, 6 PE IV) and 2 pronation-abduction injuries (1 PA II, 1 PA III). The fractures classified as III and IV are more severe than those classified as II. The right ankle was affected in 32 patients and the left in 22. All fractures had been treated with open reduction and internal fixation and all patients except one were treated with a below knee plaster cast during the first six weeks postoperatively. Weight bearing was not allowed during the plaster time. Radiographic examination of the ankle 14 months postoperatively was performed in 51 patients (three declined the investigation). The fractures were classified as healed with no displacement in 40 ankles and with slight displacement in 11 ankles. Eight patients had discrete signs of osteoarthritis (a loss of joint space of less than 50%) and two had moderate osteoarthritis (a loss of joint space of more than 50% but no bone-to-bone contact) [10]. The median Body mass index (BMI) was 26 at 14-month follow-up (IQR 3).

### Controls

Fifty-four uninjured persons, 31 men and 23 women, participated as volunteers in the age- and gender-matched control group. Each patient had a matched control and the difference in age within the pair had to be five years or less. To be included no history of neurological disease, major orthopaedic lesions, and vestibular or visual disturbance was accepted. The median age for men was 31 (IQR 26) and for women 52 years (IQR 19). The median BMI for the controls was 24 (IQR 3). In the control group only the stabilometric tests were performed. No significant difference was found between the patients and controls regarding age or BMI.

All subjects gave their informed consent to participate in the study and due to Swedish norms ethical approval was not needed for the one-year follow-up of the patients. The Research Ethics Committee at the Lund University approved the study LU 556-03 for the controls.

### Outcome measures

#### Questionnaire

Before the clinical examination a questionnaire was completed [10]. One of the questions concerned perceived functional instability of the ankle and could be answered as: (a) none, (b) 1–2/year (during exercise), (c) 1–2/month (during exercise), (d) walking on uneven ground, (e) walking on even ground, (f) constant (severe) using ankle support [24]. Before the results were analysed the patients were divided into two groups. All who had answered 'none' formed one group and the rest formed the other group.

#### Clinical examination

The 54 patients were invited and examined by the same physiotherapist (GN) a mean of 14 months (SD 1.7) post-surgery. Muscle strength was evaluated as rising-on-toes and heels. Ankle mobility was examined in loaded dorsiflexion and plantar flexion. These methods are all described in detail in Nilsson et al. [10].

**Figure 1:**
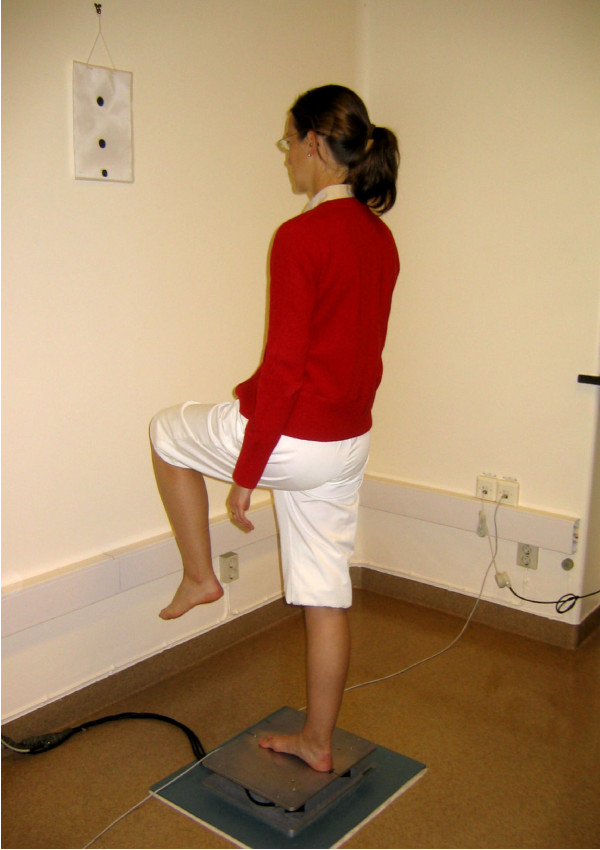
**Stabilometry in single-limb stance, tested by means of a strain gauge force plate**. A standardized position was used. The subject is a model who did not participate in the study.

Balance in single-limb stance was tested by means of a strain gauge force plate (33 × 38 cm) with the subject barefoot in a standardized position [17,18,20,25] (Figure 1). One foot was placed pointing straight forward in relation to reference lines in the frontal and sagittal planes. The other leg was flexed 90° at the hip and knee joints with both arms hanging relaxed at the sides. The subjects were instructed to stand as motionless as possible, looking straight ahead at a point on the wall 65 cm away; they were allowed to practice this position for about 20 seconds before three measurements were made on each leg, with the subjects standing alternately on their right and left leg. The mean value of these three measurements was used in the analysis.

Movements of CP in the frontal plane (FP) and in the sagittal plane (SP) were recorded for 25 seconds at a sampling frequency of 20 Hz. A sampling frequency below 2 Hz has been observed in single-limb stance in the frontal plane in uninjured subjects and individuals with perceived functional instability of the ankle [19]. We have found no reports on the frequency of CP in single-limb stance in the sagittal plane. Thus, it is reasonable to assume that a sampling frequency of 20 Hz is appropriate for measuring CP movements in single-limb stance. If single-limb balance was not maintained for 25 seconds, the results were not recorded and the measurement was repeated at a maximum of three attempts. A computer program (Viewdac 2.1, Keithley Instruments, Inc., MA, USA) was used giving the following variables: 1) average speed of CP movements in millimetres per second, and 2) number of movements exceeding 10 mm from the mean value of CP (DEV 10). The average speed is calculated as the total length of the path of CP movements divided by the test trial time and reflects the number of CP movements and the amplitude of these movements. DEV 10 is the number of movements exceeding 10 mm from the mean value of CP, reflecting the deviations of CP.

### Statistics

Statistics were produced by use of SPSS software, Version 11.5. To analyse differences between the injured and uninjured legs in the patient group and between the side-matched legs within the controls the paired Student *t*-test was used. To analyse differences between patients' injured and uninjured legs and the appropriate side-matched legs in the controls the independent Student *t*-test was used. Chi-square test, and when applicable, Fisher's exact test were used for comparisons between groups. A p-value of < 0.05 was regarded as significant. The variables rising-on-toes and heels, loaded dorsiflexion and plantar flexion were dichotomized at clinically accepted levels [26,27]. For logistic regression the continuous variables BMI and age were dichotomized, BMI at 30 kg/m^2 ^and age at 45 years. Multiple logistic regression (stepwise, backward) was performed to identify factors associated with belonging to the group unable to complete the stabilometric test. As the variables rising-on-toes and rising-on-heels were found to be intercorrelated a composite, dichotomous variable was constructed. Those who could manage to perform 25 or more rising-on-toes and 20 or more rising-on-heels formed one group (n = 30) and those who did not manage one or both of the tests formed the other group (n = 24).

## Results

### Stabilometric results versus patient characteristics and fracture characteristics

Fourteen of the 54 patients (26%), four men and ten women, could not manage to complete the 3 × 25-second single-limb stance test on the force-platform. They failed as they lost their balance and either had to put down the other foot or had to change the base of support. Patients over 45 years of age, female gender, prevalence of ankle osteoarthritis and BMI over 30 kg/m^2 ^at the 14-month follow-up to a greater extent belonged to the group that could not manage to complete the stabilometric test (Table 1). Multiple logistic regression showed that among variables that in bivariate analysis were significantly associated with managing to complete the stabilometric test (Table 1) only age over 45 years was significantly associated with not managing the test (OR = 14.80; 95%CI 2.71–74.12; p = 0.002).

### Stabilometric results versus clinical examination

Patients who were not able to complete 25 rising-on-toes (n = 23), 20 rising-on-heels (n = 18) and those who perceived functional instability of the ankle (n = 25) to a greater extent belonged to the group that did not manage to complete the stabilometric test. Ankle mobility did not influence the ability to complete the test or not (Table 2). Multiple logistic regression of functional variables showed that among variables that in bivariate analysis were significantly associated with managing to complete the stabilometric test (Table 2) the composite variable rising-on-toes and heels (the variables rising-on-toes and rising-on-heels were included as the composite variable rising-on-toes and heels) was significantly associated with not managing (OR = 14.00; 95% CI 2.71–72.36; p = 0.002). When age was included in the model this was the only variable significantly associated with not managing (OR = 15.82; 95% CI 3.04–82.37; p < 0.001). Among those 30 patients who managed to complete the 25 rising-on-toes and 20 rising-on-heels, 26 were aged under 45 years while 4 were over 45 (p < 0.001).

### Stabilometric results in patients and controls completing the test

All of the 54 persons included in the control group managed to complete the stabilometric test, whereas 40 (74%) of the 54 patients managed to complete the test (p < 0.001). The median age for these 40 patients was for men 35 years (IQR 22) and for women 42 (IQR 17). The median age for their 40 age- and gender-matched controls was for men 30 years (IQR 22) and for women 40 years (IQR 23.5). Age did not differ between the patients who managed to complete the test and their matched controls (p = 0.813). Significant differences were found between the patients' injured and uninjured legs regarding average speed in the sagittal plane with higher values in the injured leg. In the frontal plane no differences were obtained (Table 3). Between the values of the patients' injured leg and the side-matched leg of the controls no differences were observed. However, between the patients' uninjured leg and the side-matched leg of the controls the values differed significantly regarding average speed in the sagittal plane, with higher values for the controls (Table 4).

## Discussion

Out of 54 patients operated on because of an ankle fracture 40 (74%) managed to complete the single-limb stance test on a force-platform 14 months postoperatively whereas all 54 controls managed. Age over 45 years was the only risk factor significantly associated with not managing the test. When not adjusted for age, decreased strength in the ankle plantar and dorsiflexors was also a risk factor significantly associated with not managing. Comparing the patients who managed to complete the test to an age- and gender-matched control group, no differences were found between the patients' injured leg and controls concerning the stabilometric test.

Higher age is known to be related to decreased postural control reported as increased frequency of CP movements measured by stabilometric tests [28-30]. Ekdahl et al. [28] studied balance measured as single-limb stance test 3 × 30 seconds on a force-platform (AMTI) in a reference group 20–64 years of age. All subjects (54 men and 55 women) aged 20–54 managed to complete the test. In those between 55 and 64 eleven out of 43 (26%) could not manage to complete the test. In the present study, 8/13 patients over 54 years of age (62%) could not manage to complete the test, whereas all 15 patients over 54 in the control group managed to complete the test, indicating poorer results in the ankle fracture group compared with both the controls in our study and the reference group in the study by Ekdahl et al. [28]. Jonsson et al. [31] investigated 30-second one-leg stance on a force-platform (AMTI) in two groups of healthy volunteers of different ages, young adults (mean age 29.9) and elderly (mean age 70.5). All the young adults managed to complete the 30-second one-leg stance whereas only seven of the 20 persons in the elderly group managed to complete the test. Different strategies in force variability between the dynamic phase (first 5 seconds) and the static phase (last 25 seconds) and impairment to compensate for the postural disturbances caused by the weight shift and musculoskeletal weakness were supposed to be the reasons for the differences between the two groups.

In order to investigate whether any of the functional variables capable of influencing with training formed a risk factor for not managing the test, we used a regression model where age was not included. Thus, we found that decreased strength in the ankle plantar flexors and dorsiflexors, measured by number of rising-on-toes and heels, was associated with not managing to complete the stabilometric test. Several studies have been performed evaluating strength in the lower extremity and its influence on balance [32-35]. Decreased strength especially at the ankle has been reported as correlated with loss of balance [33], and in the study by Whipple et al. [32] weakness of the ankle dorsiflexors was found to be a factor underlying poor balance [32]. However, Ringsberg et al. [34] could not verify these results.

Increased sway area, measured by using a sway meter, which registered displacements of the body in 30-second periods, has been reported as one of the predictors of sustaining a fracture [11]. However, many studies have disclosed the effect of balance training in preventing recurrent injuries [16], in improving postural control [13-15,36], in reducing the feeling of "give way" [36,37] and in increasing pronator muscle strength [36]. With special attention to the elderly, exercise training with the focus on balance, gait and coordination has been found to improve static and dynamic balance [38,39].

Despite studying a small group of patients and using a method that provided limited quantitative data, but one frequently used in clinical practice, in measuring the strength in the ankle plantar flexors and dorsiflexors, we ended up with the same conclusions as reported in earlier studies, that muscle strength influences standing balance. As patients at higher ages to a higher degree have decreased balance and muscle strength even before the fracture [28,31,40] it is likely that after six weeks of immobilization they start to regain function from a lower level compared to young adults and it is therefore important to ensure that optimal rehabilitation is offered.

When looking at the patients who managed to complete the stabilometric test, a small difference was obtained between the patients' injured and uninjured legs regarding average speed in the sagittal plane. However, the mean difference was less than 10 percent, a variation that has been reported between legs in uninjured subjects [29]. The clinical relevance of these results can therefore be questioned. No differences were found between the injured leg and the controls. A study by Leanderson et al. [41], evaluating patients with acute ankle ligament sprains grade II (partial ligament rupture) and III (total ligament rupture), reported normalized balance in single-limb stance analysed by the use of stabilometry, 10 weeks post-injury compared to the uninjured side. Tropp [36] studied patients with perceived functional instability of the ankle. Ten weeks of coordination training on an ankle disc improved the stabilometry results and the improvement was significantly better than in a control group. Thirty weeks without training did not significantly change the results. In the present study postural stability was not measured until 12 months after plaster removal. Because of that no conclusion can be drawn as to whether postural stability had improved and normalized or if it had been affected at all. However, it would have been interesting to investigate the patients earlier during the process and to compare the stabilometry values over time.

## Conclusion

In conclusion, compared to an age- and gender-matched control group postural control was found to be impaired in patients over 45 years of age operated on because of an ankle fracture 14 months after surgery. Decreased strength in the ankle plantar flexors and dorsiflexors was also found to be associated with impaired postural control. In patients below 45 years no difference was found compared to their matched controls, which indicates normal postural control in single-limb stance one year after plaster removal in this age group. Further longitudinal studies are required to elucidate the development of balance performance during rehabilitation.

## Competing interests

The author(s) declare they have no competing interests.

## Authors' contributions

GN participated in the design of the study, participated in collecting the data, performed the statistical analyses, and drafted the manuscript. EA participated in the design of the study, participated in collecting the data, and in the progress and revision of the manuscript. CE participated in the design of the study, and in the progress and revision of the manuscript. ME was responsible for identifying and including the patients, and participated in the progress and revision of the manuscript. All authors read and approved the final manuscript.

## Pre-publication history

The pre-publication history for this paper can be accessed here:



## Figures and Tables

**Table 1:** Variables regarding patients who managed versus did not manage to complete the stabilometric test

	**Managed to complete n = 40**	**Did not manage to complete n = 14**	**P-value**
**Age**			<0.001
< 45 years	29	2	
≥ 45 years	11	12	

**Gender**			0.011
Men	27	4	
Women	13	10	

**Type of fracture**			0.302
Supination	27	12	
Pronation	13	2	

**Postoperative reduction results**			1.0
Congruent	32	12	
Incongruent	8	2	

**Osteoarthritis 14-month (n = 51)**			0.018
No	33	8	
Yes	4	6	

**BMI (14-month)**			0.013
< 30 kg/m2	36	8	
≥ 30 kg/m2	4	6	

**Table 2:** Bivariate associations between not being able to complete the stabilometric test and functional variables

	**Managed to complete n = 40**	**Did not manage to complete n = 14**	**P-value**
**Rising on toes (repetitions)**			< 0.001
≥ 25	29	2	
< 25	11	12	

**Rising on heels (repetitions)**			< 0.001
≥ 20	33	3	
< 20	7	11	

**Rising on toes and heels (composite variable) (repetitions)**			< 0.001
Yes #	28	2	
No ¤	12	12	

**Ankle dorsiflexion (degrees)**			0.344
≥30	23	6	
< 30	17	8	

**Ankle plantar flexion (degrees)**			0.083
≥45	25	5	
< 45	15	9	

**Percieved functional instability of the ankle**			0.028
No	25	4	
Yes	15	10	

# ≥ 25 rising on toes and ≥ 20 rising on heels

¤ < 25 rising on toes and/or < 20 rising on heels

**Table 3:** Stabilometric results in patients who managed to complete the stabilometric test 14 months postoperatively and in an age-and gender-matched control group

	**Patients**	**Patients**		**Controls**	**Controls**	
	Injured leg n = 40	Uninjured leg n = 40	P-value	Side-matched leg (injured) n = 40	Side-matched leg (uninjured) n = 40	P-value

	Mean (SD)	Mean (SD)		Mean (SD)	Mean (SD)	

**Frontal plane**						
AS (mm/s)	26.2 (6.3)	25.5 (5.5)	0.197	26.6 (6.8)	27.0 (7.4)	0.611
DEV 10 (n)	5.2 (4.2)	4.9 (2.8)	0.485	5.3 (3.9)	5.2 (4.6)	0.830

**Sagittal plane**						
AS (mm/s)	24.4 (7.2)	22.4 (5.6)	0.014	26.7 (10.6)	25.9 (6.4)	0.511
DEV 10 (n)	6.4 (4.0)	5.7 (2.7)	0.078	6.6 (4.4)	6.6 (2.9)	1.000

AS = average speed; DEV 10 = number of movements exceeding 10 mm.

**Table 4:** Stabilometric results in patients who managed to complete the stabilometric test 14 months postoperatively and in an age-and gender-matched control group

	**Patients**	**Controls**		**Patients**	**Controls**	
	Injured leg n = 40	Side-matched leg (injured) n = 40	P-value	Uninjured leg n = 40	Side-matched leg (uninjured) n = 40	P-value

	Mean (SD)	Mean (SD)		Mean (SD)	Mean (SD)	

**Frontal plane**						
AS (mm/s)	26.2 (6.3)	26.6 (6.8)	0.797	25.5 (5.5)	27.0 (7.4)	0.311
DEV 10 (n)	5.2 (4.2)	5.3 (3.9)	0.891	4.9 (2.8)	5.2 (4.6)	0.684

**Sagittal plane**						
AS (mm/s)	24.4 (7.2)	26.7 (10.6)	0.249	22.4 (5.6)	25.9 (6.4)	0.010
DEV 10 (n)	6.4 (4.0)	6.6 (4.4)	0.812	5.7 (2.7)	6.6 (2.9)	0.163

AS = average speed; DEV 10 = number of movements exceeding 10 mm
